# Reactive Oxygen Species-Induced TRPM2-Mediated Ca^2+^ Signalling in Endothelial Cells

**DOI:** 10.3390/antiox10050718

**Published:** 2021-05-03

**Authors:** Ran Ding, Ya-Ling Yin, Lin-Hua Jiang

**Affiliations:** 1 Department of Physiology and Pathophysiology, Sino-UK Joint Laboratory of Brain Function and Injury of Henan Province, Xinxiang Medical University, Xinxiang 453003, China; dingran0160@126.com (R.D.); yalingyin@xxmu.edu.cn (Y.-L.Y.); 2 School of Biomedical Sciences, Faculty of Biological Sciences, University of Leeds, Leeds LS2 9JT, UK

**Keywords:** endothelial cells, ROS, Ca^2+^ signaling, TRPM2 channel, angiogenesis, barrier dysfunction, vascular diseases

## Abstract

Endothelial cells form the innermost layer of blood vessels with a fundamental role as the physical barrier. While regulation of endothelial cell function by reactive oxygen species (ROS) is critical in physiological processes such as angiogenesis, endothelial function is a major target for interruption by oxidative stress resulting from generation of high levels of ROS in endothelial cells by various pathological factors and also release of ROS by neutrophils. TRPM2 is a ROS-sensitive Ca^2+^-permeable channel expressed in endothelial cells of various vascular beds. In this review, we provide an overview of the TRPM2 channel and its role in mediating ROS-induced Ca^2+^ signaling in endothelial cells. We discuss the TRPM2-mediated Ca^2+^ signaling in vascular endothelial growth factor-induced angiogenesis and in post-ischemic neovascularization. In particular, we examine the accumulative evidence that supports the role of TRPM2-mediated Ca^2+^ signaling in endothelial cell dysfunction caused by various oxidative stress-inducing factors that are associated with tissue inflammation, obesity and diabetes, as well as air pollution. These findings provide new, mechanistic insights into ROS-mediated regulation of endothelial cells in physiology and diseases.

## 1. Introduction

Endothelial cells form the innermost and one-cell thick layer (endothelium) of blood vessels and serve the interface between blood in the lumen and the surrounding tissues to maintain tissue homeostasis and regulate their function [1,2,3]. Important to this monolayer is that endothelial cells form tight inter-endothelial junctions that, on one hand, allow exchange of small molecules and, on the other, prevent entry of circulating leucocytes, proteins and pathological factors into the neighboring tissues to induce inflammation and damage to healthy tissue [1,2,3,4]. Reactive oxygen species (ROS), a group of radical and non-radical, oxygen-derived and chemically reactive molecules including superoxide (O_2_^•^) and hydrogen peroxide (H_2_O_2_), are a well-known regulator of endothelial cell function in physiological processes, particularly in the pathogenesis of diseases [5,6,7,8,9,10,11,12,13,14,15,16,17,18,19,20,21,22,23]. ROS, at low levels, serves physiologically important signaling molecules, for example, in angiogenesis, a process engaging proliferation and migration of endothelial cells and formation of neo-vessels from existing vessels [7]. However, generation of high levels of ROS or oxidative stress mediates detrimental effects on endothelial cells by miscellaneous pathological factors, including proinflammatory mediators that are associated with infection, high levels of glucose and free fatty acids that are linked with obesity and diabetes, and ischemia/reperfusion [8,9,10,11,12,13,14,15,16,17]. Endothelial cells are also prone to oxidative stress induced by circulating leucocytes in the blood, like neutrophils, that produce a large amount of ROS to kill invading pathogens. In short, oxidative stress is one of the important denominators, as well as a pathological hallmark, of vascular diseases and related conditions, such as hypertension and atherosclerosis, that develop in part as a result of oxidative stress-induced endothelial cell dysfunction [1,10,11,12,13,14,15,16,17,18,19,20,21,22,23], leading to the concept of antioxidant therapies [24,25,26]. In the central nervous system (CNS), endothelial cells interact with several other types of cells to constitute the neurovascular unit, an anatomically complicated and functionally vital structure that includes the blood brain barrier [27]. Oxidative stress-induced disruption of endothelial cell function and subsequent neurovascular dysfunction represent an important mechanism mediating traumatic brain damage and predisposition to vascular dementia and other neurodegenerative diseases [27,28,29,30,31,32]. Furthermore, increasing evidence suggests that alteration in endothelial cell function by oxidative stress increases the susceptibility to cardiovascular and CNS diseases and/or the severity of disease outcomes, as consequences of exposure to air pollution [33,34,35,36]. Thus, it is increasingly recognized that oxidative stress-induced endothelial cell dysfunction plays a crucial part in the pathogenesis of numerous pathologies. A better understanding of the underlying signaling mechanisms provides an opportunity to identify targets for the development of therapeutics to alleviate the debilitating impacts of such conditions.

Intracellular Ca^2+^ is the most common signaling molecule with an essential role in pleiotropic physiological functions [37,38,39]. Conceivably, disruption in intracellular Ca^2+^ homeostasis or Ca^2+^ signaling leads to of diverse pathologies. Exposure to ROS, particularly at pathologically relevant concentrations, can elevate intracellular Ca^2+^ concentration, via diverse molecular mechanisms mediating Ca^2+^ entry or Ca^2+^ release from intracellular stores such as endoplasmic reticulum (ER). Increasing evidence suggests that oxidative stress-induced Ca^2+^ signaling activates downstream Ca^2+^-dependent signaling pathways to disrupt normal cell function [40,41,42,43,44]. Transient receptor potential (TRP) melastatin 2 (TRPM2) has emerged as a key molecular mechanism enabling cells to detect ROS and respond with an increase in intracellular Ca^2+^ concentration in many physiological and pathological processes [45,46,47,48,49,50,51,52,53,54,55,56,57,58,59,60,61].

Several reviews have been published recently on the TRPM2 channel in endothelial cells in the CNS [28,53,54,55,56]. In this review, we focus on the TRPM2 channel in endothelial cells in the cardiovascular, endocrinal and respiratory systems. We begin with a brief introduction of the TRPM2 channel, followed by an overview of its expression and role in ROS-induced Ca^2+^ signaling in endothelial cells from various vascular beds. We discuss the evidence to support TRPM2-mediated Ca^2+^ signaling as an important mechanism regulating the physiological function of endothelial cells, and then its role in mediating endothelial cell dysfunction induced by various oxidative stress-inducing pathological factors associated with inflammation, obesity, diabetes and air pollution.

## 2. The Structural and Functional Properties of the TRPM2 Channel and Its Activation Mechanisms

**Figure 1:**
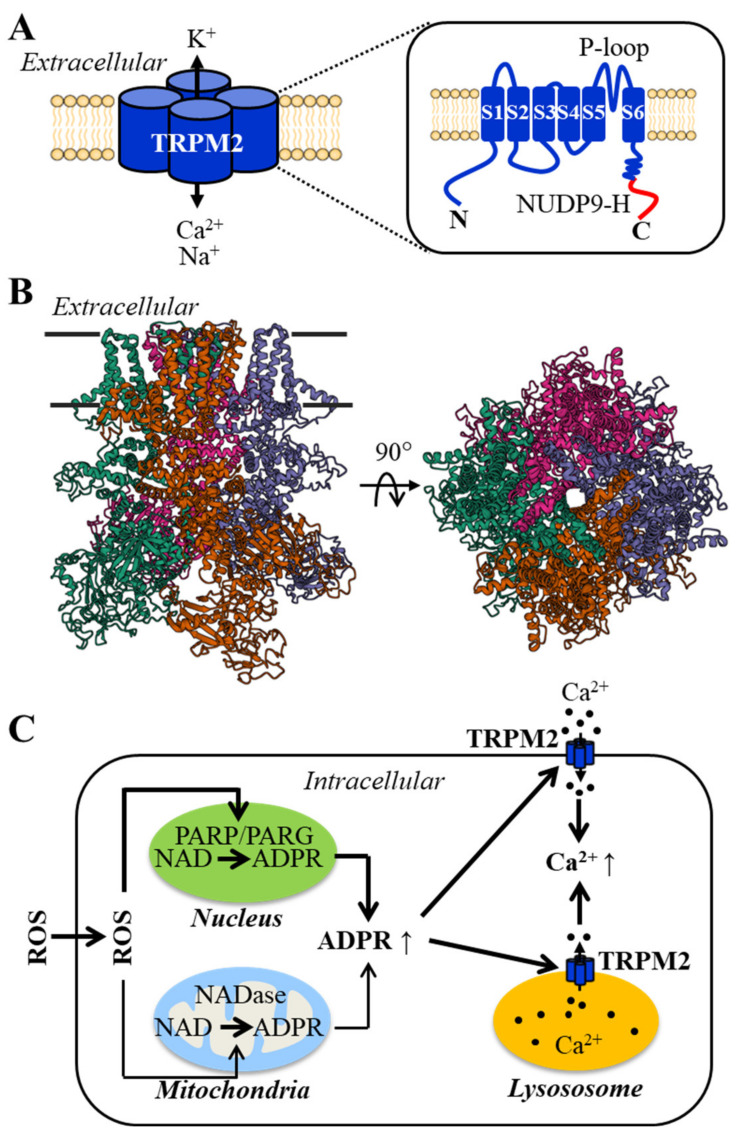
The structural properties of the TRPM2 channel and major mechanisms of channel activation by ROS. (**A**) A cartoon showing the tetrameric complex of the TRPM2 channel. Each subunit has a membrane topology of six transmembrane segments (S1–S6), with the S5 and S6 and re-entrant pore-forming loop between them (P-loop) lining the ion-conducting pore. The distal C-terminal NUDT9-H domain is engaged in ADPR binding. (**B**) The atomic structure of the human TRPM2 channel in closed state (regenerated from RXSB PDB: 6PU), viewed from parallel to the plasma membrane (**left**) or the extracellular side (**right**). Four subunits are shown in different colors. (**C**) Summary of ROS-induced activation of the TRPM2 channel. When applied extracellularly or generated extracellularly or intracellularly, ROS at high levels can induce generation of ADPR from NAD via the DNA repair mechanism in the nucleus mediated by PARP and PARG. ROS can also activate NADase in the mitochondria to generate ADPR. ADPR in turns gates the TRPM2 channel in the plasma membrane or in the lysosomes, resulting in Ca^2+^ influx or Ca^2+^ release, respectively, to elevate intracellular Ca^2+^ concentration. Abbreviations: NUDT9-H, NUDT9 homology; PARP, poly(ADPR) polymerase; PARG, poly(ADPR) glycohydrolase.

TRPM2 was initially identified as TRPC7 [62] or also formerly known as LTRPC2, the second member of the long TRPC subfamily of the transient receptor potential (TRP) superfamily [63], and has its current name, following classification of LTRPC to melastatin-related TRP or TRPM [64]. The mammalian TRPM2 channels have been well characterized molecularly and functionally and, more recently, structurally. The full-length channel proteins (TRPM2-L) are 1503 (human) or 1507 (mouse and rat) amino acid residues long, with a molecular weight of approximately 170 kDa [45,62] and have a membrane topology composed of six transmembrane segments, with the fifth and sixth segments and the re-entrant loop between them forming the ion-conducting pore, and intracellular N- and C-termini (Figure 1A). The channel is a tetrameric complex, as illustrated for the human TRPM2 channel structure (Figure 1B). The TRPM2 channel is permeable to Ca^2+^, Na^+^ and K^+^, with the cationic currents displaying a linear current/voltage (I/V) relationship [65,66]. The TRPM2 channel is gated by intracellular ADP-ribose (ADPR), intracellular Ca^2+^ or more efficiently by these two ligands working together [65,66,67,68,69]. The readers, who are interested in the structural basis for ligand binding and channel gating, can refer to recent reviews [70,71]. There is evidence to suggest that intracellular cyclic ADPR (cADPR) can activate the TRPM2 channel, albeit less potently than ADPR [72,73] (but see [74]). In addition, warm temperature (~40 °C) can activate the TRPM2 channel, and the activation threshold can be lowered to body temperature by intracellular cADPR [72]. It is worth pointing out that ADPR and cADPR can also gate ryanodine receptors, the Ca^2+^ release channels in the ER [75].

ROS, at high levels, is potent in damaging functionally important macromolecules including DNA. ADPR is long known as a byproduct of the DNA damage repair process mediated by poly(ADPR) polymerase (PARP), particularly PARP1, and poly(ADPR) glycohydrolase (PARG) in the nucleus. As such, the TRPM2 channel exhibits a high sensitivity to activation by ROS (Figure 1C) [76,77,78,79,80,81,82,83,84]. Such a ROS-induced activation mechanism has been documented in many cell types [61]. Like cADPR mentioned above, ROS can increase the thermo-sensitivity of the TRPM2 channel by oxidizing a methionine residue in the N-terminus [83]. There is also evidence to suggest that ROS can induce TRPM2 channel activation through NADase-mediated ADPR generation in the mitochondria (Figure 1C) [85]. The TRPM2 channel is thus one of the redox-sensitive TRP channels [86,87,88,89].

The TRPM2 channel is expressed in a variety of cell types, mainly on the cell surface and also or exclusively in the membranes of intracellular organelles, such as lysosomes in pancreatic-β and dendritic cells [90,91,92]. In addition to TRPM2-L, several alternative splicing isoforms have been identified, including the short TRPM2 or TRPM2-S [45]. The TRPM2-S isoform only contains the N-terminus and first two transmembrane segments and, therefore, cannot form an ion channel on its own, but its overexpression has been demonstrated to inhibit the channel formed by the TRPM2-L [80,82]. Studies have disclosed important physiological roles for the TRPM2 channel, including insulin secretion [49], immune responses [47,50] and warmth sensing [93,94], and also support a critical role for the TRPM2 channel, particularly TRPM2-mediated Ca^2+^ signaling in linking a diversity of ROS-inducing factors to associated pathologies, including ischemic stroke, neurodegenerative diseases, inflammatory diseases, cancer and diabetes [45,46,47,48,49,50,51,52,53,54,55,56,57,58,59,60,61].

## 3. TRPM2 Channel Expression in Endothelial Cells and Its Role in ROS-Induced Ca^2+^ Signaling

The TRPM2 channel in endothelial cells has been examined at mRNA, protein and functional levels in cell preparations from different species and vascular beds. TRPM2 channel expression in endothelial cells was first examined in human pulmonary artery endothelial cells [82]. TRPM2 mRNA transcripts and proteins were detected, using reverse transcription-polymerase chain reaction (RT-PCR) and Western blotting, respectively. Consistently, intracellular application of non-hydrolysable 3-deaza-cADPR evoked an inward current. H_2_O_2_ is widely used in characterizing ROS-induced TRPM2 channel activation. Exposure to H_2_O_2_ (300 μM) induced a cationic current with a linear I/V relationship. H_2_O_2_-induced current was reduced by treatment with TRPM2-specific siRNA, as well as treatment with DPQ, a PARP inhibitor. Exposure to H_2_O_2_ also induced Ca^2+^ influx, which was suppressed by treatment with TRPM2-siRNA or an anti-TRPM2 blocking antibody, overexpression of TRPM2-S, or treatment with DPQ or 3-AB, another PARP inhibitor. These results are combined to support the TRPM2 channel as a major mechanism mediating ROS-induced Ca^2+^ influx in endothelial cells [82]. A similar finding has been reported in human lung microvascular endothelial cells [95]. TRPM2 expression was shown using Western blotting. Exposure to H_2_O_2_ (300 μM) induced ADPR generation and Ca^2+^ influx, which were attenuated by treatment with PARP1-siRNA. H_2_O_2_-induced Ca^2+^ response was also reduced by treatment with TRPM2-specific siRNA, or overexpression of the TRPM2-C1006A mutant, which forms a non-functional channel on its own but imposes a dominant negative inhibition of the TRPM2 channel co-assembled with the wild-type protein [96]. Therefore, in human lung microvascular endothelial cells, the TRPM2 channel mediates H_2_O_2_-induced Ca^2+^ influx, following PARP1-dependnet ADPR generation [95]. In human umbilical vein endothelial cells (HUVECs), TRPM2 was also detected using RT-PCR in an early study [97] and, consistently, exposure to H_2_O_2_ (3 mM) elevated intracellular Ca^2+^ concentration that was attenuated by treatment with TRPM2-specific siRNA [98]. In mouse aortic endothelial cells, exposure to H_2_O_2_ induced a cationic current with a linear I/V relationship and an increase in intracellular Ca^2+^ concentration, and both current and Ca^2+^ responses were suppressed with treatment with N-(p-amylcinnamoyl)anthranilic acid (ACA), a non-selective TRPM2 channel inhibitor. These results support an important role of the TRPM2 channel in mediating ROS-induced Ca^2+^ influx in mouse aortic endothelial cells [99]. In mouse lung microvascular endothelial cells, TRPM2 expression was demonstrated using Western blotting, but the channel function and its role in H_2_O_2_-induced Ca^2+^ signaling were not established [100].

TRPM2 channel expression has been also documented in endothelial cell lines. In H5V cells, a mouse heart micro-vessel endothelial cell line, TRPM2 expression was detected using Western blotting [101]. Exposure to H_2_O_2_ (3 mM) induced a cationic current with a linear I/V relationship and Ca^2+^ influx, which were attenuated by treatment with TRPM2-specific siRNA or an anti-TRPM2 blocking antibody [101]. Similarly, in bEND.3 cells, a mouse brain endothelial cell line, TRPM2 expression was shown using Western blotting [84]. In addition, intracellular application of ADPR induced a cationic current, with a linear I/V relationship and exhibiting the sensitivity to inhibition by ACA or 2-aminoethoxydiphenyl borate (2-APB), another non-selective TRPM2 channel inhibitor. Intracellular application of amyloid β40 (Aβ40) peptide (300 nM) also evoked a cationic current with the same biophysical properties as the ADPR-induced current. Aβ40-induced current was inhibited by treatment with TRPM2-specific siRNA, ACA or 2-APB, and by treatment with PJ34, a PARP inhibitor, or ADP-HPD, a PARG inhibitor. Aβ40-induced current was also inhibited by treatment with gp91ds-tat, a peptide inhibitor of NADPH oxidases (NOX) that the catalyse generation of superoxide, or treatment with MnTBAP, a ROS scavenger. Exposure to H_2_O_2_ (500 μM) induced Ca^2+^ influx that was inhibited by treatment with ACA [84]. Likewise, exposure to Aβ40 raised the intracellular Ca^2+^ concentration that was attenuated by treatment with TRPM2-specific siRNA, ACA or 2-APB. Taken together, these results provide evidence to show an important role for the TRPM2 channel in mediating Ca^2+^ signaling induced by Aβ40 via NOX-mediated ROS generation and subsequent PARP/PARG-mediated ADPR generation [84].

In summary, a number of studies show that TRPM2 is expressed as a plasma-membrane Ca^2+^-permeable channel and plays a key role in mediating ROS-induced Ca^2+^ signaling in endothelial cells.

## 4. TRPM2 Channel in VEGF-Induced Angiogenesis and Post-Ischemic Neovascularization

Blood vessel formation is driven by both angiogenesis and arteriogenesis, with the latter mainly responsible for enlargement and remodeling of existing collateral arteries and formation of conductance vessels [1,2,3,102,103,104]. Neovascularization represents an adaptive response to ischemia/reperfusion-induced vascular damage, engaging both angiogenesis and arteriogenesis [105]. It is well-established that vascular endothelial growth factor (VEGF), by ligation of its receptor VEGFR2, induces multiple signaling pathways in endothelial cells to drive angiogenesis and post-ischemic neovascularization. For example, there also exists evidence that VEGF induces Ca^2+^-release activated channel-mediated Ca^2+^ signaling [106]. VEGF induces angiogenesis and neovascularization of ischemic tissues via NOX2-mediated ROS [107,108] and VEGF-induced ROS generation results in activation of proto-oncogene tyrosine kinase c-Src to phosphorylate vascular endothelial (VE)-cadherin and thereby to promote VE-cadherin internalization and disassembly of adherens junctions, a critical step triggering angiogenesis [109,110].

**Figure 2:**
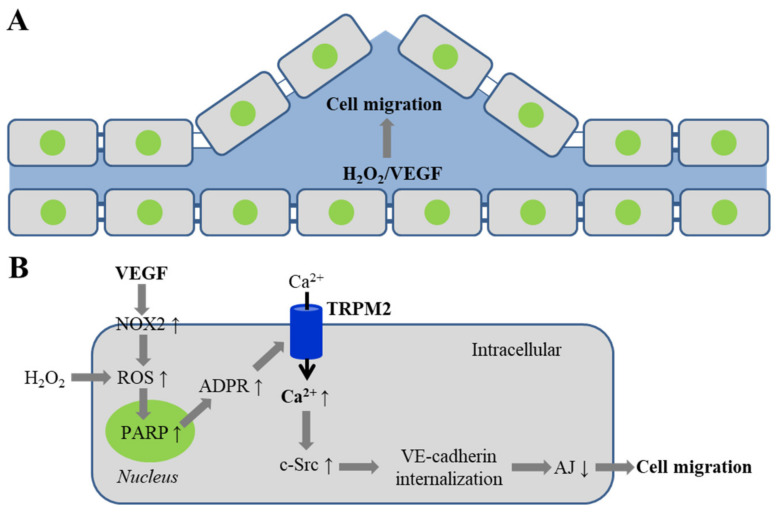
TRPM2-mediated Ca^2+^ signaling in VEGF-induced endothelial cell migration. VEGF induces endothelial cell migration (**A**) through TRPM2-mediated Ca^2+^ signaling mechanism in (**B**). VEGF induces NOX2-mediated generation of ROS that (or exposure to H_2_O_2_) activates PARP to generate ADPR and the TRPM2 channel. TRPM2-mediated Ca^2+^ influx activates c-Src kinase, which phosphorylates VE-cadherin to promote its internalization and disassembly of AJ. Abbreviations: VEGF, vascular endothelial growth factor; NOX2, NADPH oxidase 2; PARP, poly(ADPR) polymerase; VE, vascular endothelial; AJ, adherens junctions.

Mittal et al. examined the role of TRPM2-mediated Ca^2+^ signaling in VEGF-induced endothelial cell migration and angiogenesis [111]. Exposure of human pulmonary artery endothelial cells to VEGF (50–150 ng/mL) induced ROS generation, monitored using 2′,7′-dichlorodihydrofluorescein diacetate (H2DCFDA), a fluorescent indicator of ROS, and also using Amplex Red assay. Exposure to VEGF (50 ng/mL) also led to an increase in intracellular Ca^2+^ concentration. Such Ca^2+^ response was inhibited by treatment with apocynin, a generic NOX inhibitor, or catalase, a H_2_O_2_ scavenger, 3-ABA, a PARP inhibitor or overexpression of the TRPM2-C1006A mutant. Similarly, in mouse lung endothelial cells, exposure to VEGF induced an increase in intracellular Ca^2+^ concentration, with the Ca^2+^ response being significantly smaller in cells from TRPM2-KO mice than in cells from WT mice. These results show that VEGF induces NOX-mediated generation of ROS and in turn PARP-dependent activation of the TRPM2 channel and Ca^2+^ influx in endothelial cells (Figure 2). As shown using co-immunoprecipitation and proximal ligation assay, exposure to VEGF (70 ng/mL) induced the TRPM2 channel to interact first with c-Src kinase and then with VE-cadherin to form a signaling complex. VEGF induced c-Src activation earlier than VE-cadherin phosphorylation and, likewise, exposure to H_2_O_2_ (300 μM) induced c-Src activation earlier than VE-cadherin phosphorylation, all of which were attenuated by treatment with TRPM2-specific siRNA or overexpression of the TRPM2-C1006A mutant. Consistently, VEGF induced a lower level of c-Src activation and VE-cadherin phosphorylation in lung endothelial cells from TRPM2-KO mice than WT mice. VEGF-induced c-Src activation and VE-cadherin phosphorylation were reduced by treatment with apocynin, and VEGF-induced VE-cadherin phosphorylation was attenuated by treatment with NOX2-specific siRNA, 3-ABA or DPQ. In addition, VEGF stimulated endothelial cell migration, shown using the wound healing assay, and also induced a full recovery of endothelial barrier function that was impaired by an electric shock, by measurement of trans-endothelial electrical resistance (TER), a sensitive indicator of endothelial permeability. VEGF-induced endothelial cell migration and recovery of endothelial barrier function were inhibited by treatment with TRPM2-specific siRNA, as well as c-Src-specific siRNA. The study further examined the role of the TRPM2 channel in VEGF-induced angiogenesis, using in vivo Matrigel plug assay, in which the plugs were supplemented with VEGF (100 ng/mL), implanted subcutaneously in adult male C57BL/6 mice and examined 10 days later, and also using ex vivo mouse aortic ring assay [111]. VEGF stimulated angiogenesis and vessel formation in the plugs in WT mice, and promoted capillary sprouting and tube formation in aortic rings from WT mice. These VEGF-induced in vivo and ex vivo effects were reduced in TRPM2-KO mice or aortic rings from TRPM2-KO mice. Collectively, these results suggest that TRPM2-mediated Ca^2+^ signaling is critical in coupling VEGF-induced NOX2-mediated ROS generation to c-Src activation, VE-cadherin phosphorylation and internalization, and disassembly of adherens junctions that assists cell migration (Figure 2B).

The same study also examined the role of the TRPM2 channel in neovascularization in mice after introduction of hindlimb ischemia via femoral artery ligation [111]. The blood flow in ischemic limbs in WT mice was recovered faster and better after 28 days post-ischemia than in chimeric TRPM2-KO mice, namely, TRPM2-KO mice with prior transplanted with bone marrow from WT mice to restore the TRPM2 expression in immune cells. The wall thickness and lumen diameter of arterial vessels were also reduced in chimeric TRPM2-KO mice compared to those in WT mice. In addition, there were fewer CD31-positive capillaries and α-smooth muscle actin-positive arterioles in gastrocnemius muscles from chimeric TRPM2-KO mice than WT mice, examined 28 days after ischemia. Interestingly, strong TRPM2 expression in newly formed capillaries was observed using immunostaining. These results support the importance of TRPM2-mediated Ca^2+^ signaling in angiogenesis and post-ischemic neovascularization [111].

In summary, TRPM2-mediated Ca^2+^ signaling in endothelial cells plays an important role in mediating VEGF-induced angiogenesis and post-ischemic neovascularization.

## 5. TRPM2 Channel in Endothelial Dysfunction by Oxidative Stress-Inducing Pathological Factors

In this section, we discuss studies that show a role for TRPM2-mediated Ca^2+^ signaling in ROS-induced disruption of endothelial function by diverse oxidative stress-inducing pathological factors associated with inflammation, obesity, diabetes, and air pollution.

### 5.1. Endothelial Barrier Dysfunction via Inducing Cell Death

**Figure 3:**
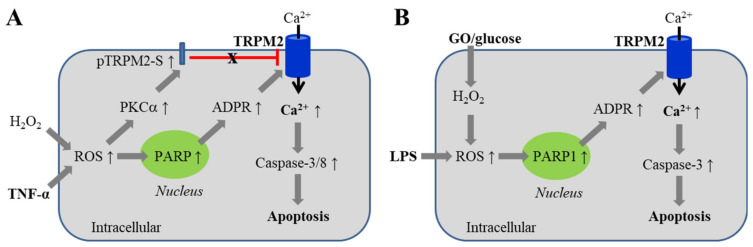
TRPM2-mediated Ca^2+^ signaling in endothelial cell death induced by ROS-inducing factors. (**A**) Generation of ROS induced by TNF-α (or exposure to H_2_O_2_) activates PARP to generate ADPR and the TRPM2 channel to induce Ca^2+^ influx. In addition, ROS activates PKCα and promotes its interaction with and thereby phosphorylation of TRPM2-S, leading to disassociation of TRPM2-S with, and disinhibition of, the TRPM2 channel. TRPM2-mediated Ca^2+^ influx activates caspase-3 and caspase-8, triggering apoptosis. (**B**) Generation of ROS by LPS, or generation of H_2_O_2_ by GO in the presence of glucose activates PARP1 to generate ADPR and the TRPM2 channel to mediate Ca^2+^ influx, resulting in activation of caspase-3 and apoptosis. Abbreviations: TNF-α, tumor necrosis factor-α; PARP, poly(ADPR) polymerase; PKC, protein kinase C; GO, glucose oxidase; LPS, lipopolysaccharide.

It is well known that oxidative stress mediates endothelial cell death, particularly apoptotic cell death, induced by various pathological factors, e.g., lipopolysaccharide (LPS), a bacterial endotoxin evoking inflammation, and tumor necrosis factor (TNF)-α, a major proinflammatory cytokine, both of which can stimulate mitochondrial and NOX-mediated ROS generation [8,112,113]. Oxidative stress-mediated endothelial cell death contributes to endothelial barrier function [10], leading to vascular tissue inflammation and damage. Such a mechanism is strongly implicated in the pathogenesis of atherosclerosis [19]. One of the well-established roles for the TRPM2 channel is to mediate oxidative stress-induced cell death [61]. The role for the TRPM2 channel in mediating oxidative stress-induced endothelial cell death was firstly demonstrated in H5V cells [101]. Exposure to H_2_O_2_ at concentrations (156–2500 μM) for 1 h resulted in a concentration-dependent reduction in cell viability, determined using MTT assay. Prolonged exposure to H_2_O_2_ (3 mM for 6–24 h) led to activation of caspase-3 and caspase-8, DNA fragmentation, and nuclear condensation and fragmentation, consistent with cell death via apoptosis. These effects were attenuated by treatment with TRPM2-specific siRNA or an anti-TRPM2 blocking antibody. Similarly, exposure to TNF-α (10 ng/mL) for 36 h decreased endothelial cell viability that was also prevented by treatment with TRPM2-specific siRNA or an anti-TRPM2 blocking antibody [101]. These results support a role for the TRPM2 channel in mediating oxidative stress-induced endothelial cell death (Figure 3A).

The role of the TRPM2 channel in mediating oxidative stress-induced apoptotic cell death has been investigated in human pulmonary artery endothelial cells and mouse lung endothelial cells [114]. Exposure to H_2_O_2_ at pathologically relevant concentrations (25–300 μM) for 24 h induced a concentration-dependent increase in apoptotic cell death, based on analysis of staining with annexin V-phycoerythrin and 7-aminoactinomycin D. Similar apoptotic cell death was observed after exposure to glucose oxidase in the presence of glucose (GO/glucose), a condition known to generate H_2_O_2_. Apoptotic cell death induced by H_2_O_2_ or GO/glucose was attenuated by treatment with TRPM2-specific siRNA or an anti-TRPM2 blocking antibody. Consistently, treatment of WT mice with H_2_O_2_ or GO/glucose induced apoptosis of endothelial cells in lung vessels, and caspase-3 activation and apoptosis-associated PARP cleavage in lung tissues, all of which were reduced in TRPM2-KO mice. Challenging WT mice with intraperitoneal injection of LPS to mimicking infection resulted in apoptosis of endothelial cells in lungs, assessed 4 h after LPS injection by staining of frozen lung tissues using a VE-cadherin antibody in combination with terminal deoxynucleotidyl transferase dUTP nick end labeling (TUNEL) assay. LPS-induced endothelial cell death was almost absent in chimeric TRPM2-KO mice. Furthermore, WT mice poorly survived following LPS injection, but the survival rate of chimeric TRPM2-KO mice was significantly improved. These results support an important role of the TRPM2 channel in oxidative stress-induced endothelial cell apoptotic death (Figure 3B) and suggest that such cell death increases the susceptibility to pulmonary inflammation and lung damage [114]. As shown in a recent study, mice with endothelial cell-specific TRPM2-KO showed an improved survival rate after LPS injection, but a different mechanism has been proposed to underlie such a beneficial effect [95].

It is shown in several cell types, including endothelial cells, that the overexpression of TRPM2-S can inhibit ROS-induced TRPM2-mediated effects. The study discussed above [114] has revealed a mechanism underlying the inhibition by TRPM2-S of oxidative stress-induced TRPM2-mediated Ca^2+^ response and apoptotic cell death in human pulmonary artery endothelial cells (Figure 3A). In resting cells, TRPM2-S interacts with TRPM2-L. Exposure to H_2_O_2_ or TNF-α activates protein kinase C-α (PKCα) and induces PKCα to interact with and phosphorylate TRPM2-S and, upon phosphorylation, TRPM2-S disassociates with TRPM2-L, leading to disinhibition of the TRPM2 channel. In other words, oxidative stress induces disinhibition by TRPM2-S of the TRPM2 channel, as well as PARP-dependent activation of TRPM2 channel. As shown in the same study, LPS-induced endothelial cell apoptosis in lungs was also lessened, and the survival rate was improved following LPS injection in chimeric PKCα-KO mice, as observed in chimeric TRPM2-KO [114], which further supports that oxidative stress-induced PKCα-dependent disinhibition of the TRPM2 channel by TRPM2-S is important in TRPM2-mediated endothelial cell death and lung damage that are associated with inflammation.

### 5.2. Endothelial Barrier Dysfunction via Disrupting Inter-Endothelial Junctions

**Figure 4:**
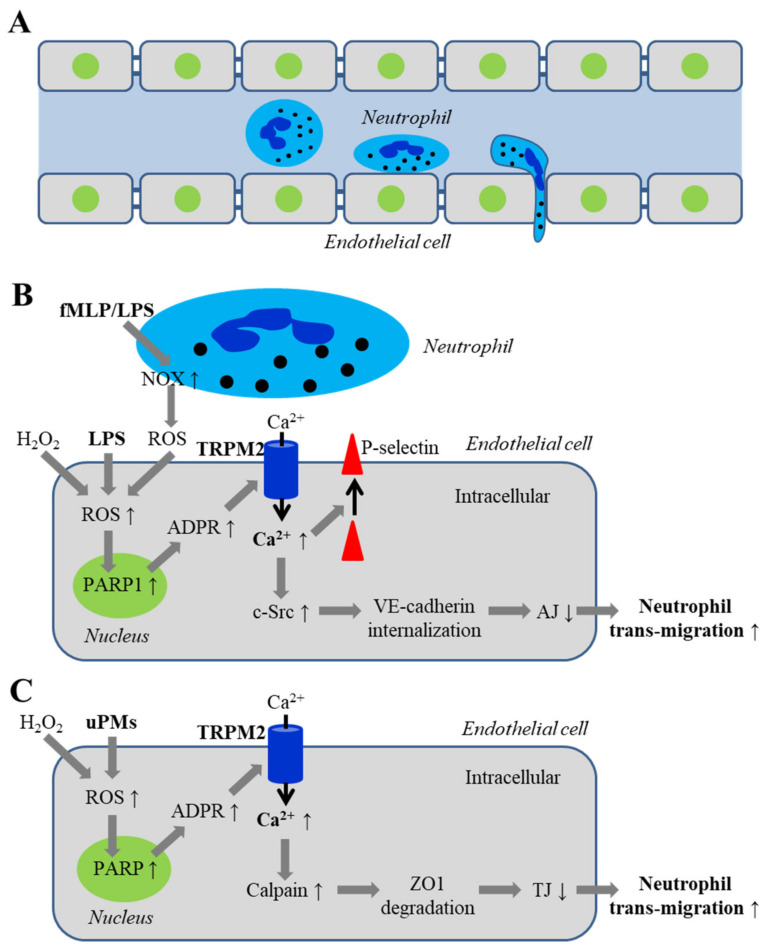
TRPM2-mediated Ca^2+^ signaling in endothelial barrier dysfunction and neutrophil trans-migration associated with inflammation and exposure to ultrafine particulate matters. (**A**) The cartoon illustrates attachment of neutrophils to endothelial cells and trans-endothelial migration through signaling mechanisms in response to tissue infection (**B**) or exposure to uPMs (**C**). (**B**) ROS generated by LPS-exposed endothelial cells (or exposure to H_2_O_2_) or generated via NOX in fMLP/LPS-treated neutrophils and released to endothelial cells, activates PARP1 and the TRPM2 channel. TRPM2-mediated Ca^2+^ influx induces c-Src activation and VE-cadherin phosphorylation to promote its internalization and disassembly of AJ, facilitating trans-endothelial migration of neutrophils. TRPM2-mediated Ca^2+^ influx also increase cell surface expression of P-selectin to recruit neutrophils. (**C**) ROS generated by uPM-exposed endothelial cells (or exposure to H_2_O_2_) activates PARP and the TRPM2 channel. TRPM2-mediated Ca^2+^ influx induces calpain activation, ZO1 protein degradation and loss of TJ, facilitating trans-endothelial migration of neutrophils. Abbreviations: fMLP, N-formylmethionyl-leucyl-phenylalanine; LPS, lipopolysaccharide; NOX, NADPH oxidase; PARP, poly(ADPR) polymerase; VE, vascular endothelial; AJ, adherens junctions; uPM, ultrafine particulate matters; ZO1, zona occludens-1; TJ, tight junctions.

Endothelial barrier function, as introduced above, is crucial in protecting healthy vascular tissues. It is well documented that oxidative stress can impair endothelial barrier function via promoting Ca^2+^ influx into endothelial cells to disrupt inter-endothelial tight junctions [10,41]. Neutrophils are the most abundant circulating leukocytes and act as the first-line defense against tissue infection by generating a large amount of ROS to destroy invading pathogens. However, ROS generated by neutrophil can disrupt endothelial permeability that facilitates trans-endothelial migration of neutrophils and, as a result, risks tissue damage. There is increasing evidence to suggest an important role for TRPM2-mediated Ca^2+^ signaling in mediating endothelial barrier dysfunction leading to increased trans-endothelial migration of neutrophils in response to various ROS-inducing pathological factors (Figure 4).

#### 5.2.1. Disruption of Adherens Junctions by ROS and Oxidative Stress Associated with Infection

Malik and colleagues were the first to investigate the role of TRPM2-mediated Ca^2+^ signaling in H_2_O_2_-induced endothelial barrier dysfunction using human pulmonary artery endothelial cells [82]. Exposure of endothelial cell monolayers to 300 μM or higher concentrations of H_2_O_2_ impaired endothelial barrier function indicated by a reduction in TER. H_2_O_2_-induced barrier function was attenuated by treatment with TRPM2-specific siRNA or an anti-TRPM2 blocking antibody, overexpression of TRPM2-S, or by treatment with 3-AB or DPQ. These results support the importance of PARP-dependent TRPM2 channel activation in H_2_O_2_-induced endothelial barrier dysfunction. As shown in a recent study described above, exposure of human lung microvascular endothelial cells to H_2_O_2_ promoted PARP1-dependent ADPR generation and TRPM2 channel activation, leading to Ca^2+^ influx, c-Src activation and VE-cadherin phosphorylation, promoting VE-cadherin internalization and disassembly of adherens junctions [95]. H_2_O_2_-induced c-Src activation, VE-cadherin phosphorylation and, furthermore, endothelial barrier dysfunction, were attenuated by treatment with TRPM2-specific siRNA or PARP1-specific siRNA [95]. These studies altogether support that oxidative stress-induced TRPM2 channel activation and TRPM2-mediated Ca^2+^ signaling promote c-Src activation, VE-cadherin phosphorylation and internalization, and disassembly of adherens junctions, leading to reduced endothelial barrier function (Figure 4B).

The recent study has also examined the role of the TRPM2 channel in LPS-induced disruption of endothelial barrier function, trans-endothelial migration of neutrophils and the consequence on the survival of mice [95]. In WT mice, intraperitoneal injection with LPS (10 mg/kg) led to increased lung vascular permeability, and trans-endothelial migration of neutrophils, shown by determining the myeloperoxidase activity in lung tissues and live imaging neutrophils in micro-vessels and alveoli of lungs and also using hematoxylin and eosin (H&E) staining to reveal vascular infiltration of leucocytes. These effects were strongly reduced or absent in endothelial cell-specific TRPM2-KO mice. LPS-induced trans-endothelial migration of neutrophils in lungs of WT mice was reduced by treatment with PARP1-specific siRNA or overexpression of the TRPM2-C1006A mutant. Conversely, in TRPM2-KO mice, the deficiency in the LPS-induced trans-endothelial migration of neutrophils was rescued by injection with TRPM2 cDNA to restore the TRPM2 expression. In addition, injection with LPS (20 mg/mL) in WT mice induced edema formation in lungs, and all mice died in less than 96 h. However, edema formation induced by such a lethal dose of LPS was suppressed in TRPM2-KO mice and, importantly, a majority of TRPM2-KO mice survived even 96 h after LPS injection [95]. These findings provide compelling evidence for an important role of the TRPPM2 channel in endothelial cells in mediating LPS-induced trans-endothelial migration of neutrophils, vascular damage and death.

As further examined in vitro [95], exposure to mouse neutrophils stimulated with N-formylmethionyl-leucyl-phenylalanine (fMLP), a potent leukocyte chemotactic factor, induced Ca^2+^ influx in human lung microvascular endothelial cells. The Ca^2+^ response was attenuated by treatment with PARP1-specific or TRPM2-specific siRNA, or by overexpression of the TRPM2-C1006A mutant. Such neutrophil-induced Ca^2+^ response was much smaller in endothelial cells exposed to mouse neutrophils treated with DPI, a NOX inhibitor, or NOX-deficient neutrophils isolated from gp91Phox-KO mice. These results suggest that ROS, generated via NOX and released by neutrophils, sufficiently induce TRPM2 channel activation in juxtaposed endothelial cells. Mouse neutrophils treated with fMLP, like with H_2_O_2_, induced c-Src activation and VE-cadherin phosphorylation in endothelial cells, which were reduced by treatment of endothelial cells with TRPM2-specific siRNA. Consistently, migration of fMLP-treated neutrophils through endothelial cell monolayers was attenuated by treatment of endothelial cells with TRPM2-specific siRNA. Such trans-endothelial migration was also reduced for DPI-treated mouse neutrophils or gp91 Phox-KO mouse neutrophils. Furthermore, LPS-induced trans-endothelial migration of neutrophils in WT mice, in which endogenous neutrophils were depleted and replenished by gp91 Phox-KO neutrophils, was reduced compared to that in WT mice that were replenished with WT neutrophils. LPS injection also induced c-Src activation and VE-cadherin phosphorylation in WT mice, which were attenuated in TRPM2-KO mice. These results endorse the notion that neutrophils generate ROS via NOX to activate the TRPM2 channel in endothelial cells in direct contact or in close vicinity, thereby increasing the permeability of inter-endothelial junction to facilitate trans-endothelial migration of neutrophils. In addition, the cell surface expression of P-selectin [95], which is important for recruitment of neutrophils to endothelial cells, was enhanced in endothelial cells after exposure to fMLP-treated neutrophils and such neutrophil-induced upregulation of cell surface expression of P-selectin was decreased in endothelial cells treated with TRPM2-specific siRNA. In short, the TRPM2 channel in endothelial cells mediates dual mechanisms that work together to cause vascular damage in response to infection. On one hand, activation of the TRPM2 channel increases the permeability of endothelial barrier to facilitate trans-endothelial migration of neutrophils by activation of c-Src, phosphorylation and internalization of VE-cadherin and disassembly of adherens junctions and, on the other, recruits neutrophils by upregulating the cell surface P-selectin expression in endothelial cells (Figure 4A,B).

#### 5.2.2. Disruption of Inter-Endothelial Tight Junctions Induced by Airborne Fine Particulate Matters

Air pollution has been increasingly recognized as one major health risk factor, because a mounting number of epidemiological and laboratory animal-based studies exist to support a causative relationship of chronic exposure to air pollution with increased incidence, severity and mortality of respiratory, cardiovascular and even CNS diseases [33,34,35,36,115,116]. Airborne fine particulate matters (PMs) with an aerodynamic diameter of <2.5 μm, particularly ultrafine PMs (uPMs) of <200 nm and nanometer-sized PMs or nanoparticles, have attracted growing attention for their potential harmful effects, because these tiny PMs can travel deep into the airways and lungs, penetrate into the blood circulation and even reach the brain [33,116,117,118]. Moreover, it is known that the smaller the size of PMs, the more capable of causing oxidative stress they become, and the more severe their cytotoxicity is [33].

A recent study has drawn attention to the role of the TRPM2 channel in endothelial barrier dysfunction, pulmonary inflammation and lung damage induced by uPMs of 100–300 nm collected from ambient air [100]. Exposure of human lung microvascular endothelial cell monolayers to uPMs (10–100 μg/mL) resulted in a concentration-dependent reduction in TER and increase in the permeability to dextran, demonstrating endothelial barrier dysfunction. Exposure to uPMs (100 μg/mL) for 1–6 h reduced the levels of zona occludens-1 (ZO-1) and ZO-2 proteins, which are known to participate in the formation of inter-endothelial tight junctions. Both uPMs-induced endothelial barrier dysfunction and loss of ZO-1 protein were attenuated by treatment with N-acetylcysteine (NAC), an antioxidant, or EUK-134, a ROS scavenger. Exposure to uPMs also induced activation of calpain, a Ca^2+^-sensitive protease, and ZO-1 protein degradation. In addition, exposure to uPMs elevated intracellular Ca^2+^ concentration, which was inhibited by treatment with TRPM2-specific siRNA or an anti-TRPM2 blocking antibody, or by treatment with NAC or 3-AB, indicating that exposure to uPMs induces ROS generation and PARP-dependent TRPM2 channel activation. Moreover, uPMs-induced endothelial barrier function and loss of ZO-1 protein were considerably rescued by treatment with TRPM2-specific siRNA or an anti-TRPM2 blocking antibody. These results support the important role of TRPM2-mediated Ca^2+^ signaling in coupling uPMs-induced ROS generation to calpain activation, ZO-1 degradation and endothelial barrier dysfunction (Figure 4C).

Consistent with the results from human lung microvascular endothelial cells, the same study further showed that intra-tracheal aspiration of uPMs (10 mg/kg in 50-μL saline) led to several effects in adult AJ male mice, including loss of the ZO-1 protein in lung tissues, increased levels of total proteins and proinflammatory cytokines, interleukin (IL)-6 and TNF-α and more leucocytes in bronchoalveolar lavage fluids, and lung tissue damage. These effects were alleviated by intra-peritoneal injection with NAC or calpeptin, a calpain inhibitor, or by overexpression of ZO-1 in lung tissues. These results further support the importance of the ROS/calpain/ZO-1 pathway in uPMs-induced endothelial barrier dysfunction, pulmonary inflammation and lung tissue damage [100]. However, the study was short of evidence to validate the role of the TRPM2 channel in uPMs-induced pathological effects in vivo. A distinctive or additional TRPM2-mediated cellular mechanism have been proposed for silico nanoparticles-induced pulmonary inflammation and lung tissue damage in adult male C57BL/6 mice in another recent study [119]. Namely, activation of the TRPM2 channel in epithelial cells disrupts intracellular Ca^2+^ and Zn^2+^ homeostasis, leading to generation of proinflammatory cytokines, IL-1β and IL-6, and chemokines, CXCL-1 and CXCL-6 to recruit leucocytes, which together cause pulmonary inflammation and lung tissue damage [119].

In summary, increasing evidence supports TRPM2-mediated Ca^2+^ signaling as an important mechanism in oxidative stress-induced endothelial barrier dysfunction and trans-endothelial migration of neutrophils that induces pulmonary inflammation and lung damage.

### 5.3. Obesity-Associated Endothelial Insulin Resistance

Compelling evidence exists to suggest that oxidative stress due to ROS generation plays a significant part in the development of obesity-associated endothelial insulin resistance that contributes to the pathogenesis of cardiovascular and metabolic diseases, such as atherosclerosis, hypertension and type-2 diabetes [11,18,19,20,21,22,120,121]. A recent study has investigated the role of the TRPM2 channel in mediating obese-associated endothelial insulin resistance [99]. TRPM2 expression in mouse aortic endothelial cells was progressively elevated in cells isolated from mice and also in sliced mouse aorta from adult male C57BL/6J mice after 16 weeks of feeding with a high-fat diet (HFD). Consistently, H_2_O_2_-induced currents and Ca^2+^ influx that were sensitive to inhibition by ACA were significantly greater in endothelial cells isolated from HFD-fed mice than in cells from mice fed with low-fat chow diet (LFD). These results indicate upregulation of TRPM2 channel expression in endothelial cells in obese mice. Furthermore, insulin-induced endothelium-dependent relaxation of aorta from HFD-fed mice was weakened as compared LFD-fed mice. Such deficient vasorelaxation was in part rescued by treatment of aorta with ACA, suggesting a role for the TRPM2 channel in obesity-associated endothelial insulin resistance [99].

**Figure 5:**
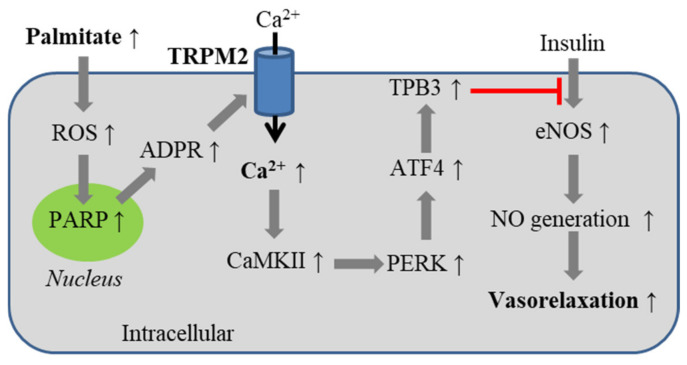
TRPM2-mediated Ca^2+^ signaling in endothelial insulin resistance induced by palmitate associated with obesity. Exposure to palmitate induces ROS generation and PARP-dependent activation of the TRPM2 channel. TRPM2-mediated Ca^2+^ influx induces activation of CaMKII and PERK and expression of ATF4 and TPB3, leading to the inhibition of insulin-induced vasorelaxation that depends on eNOS-mediated generation of NO. Abbreviations: PARP, poly(ADPR) polymerase; CaMKII, Ca/calmodulin-dependent kinase II; TRB3, pseudo-kinase tribble 3; eNOS, endothelial NO synthase; NO, nitric oxide.

It is well established that nitric oxide (NO) is a key signaling molecule inducing endothelium-dependent vasorelaxation [120]. Palmitate, a circulating free fatty acid with its concentration significantly elevated in obese conditions, can stimulate NOX-mediated ROS generation to inhibit insulin-induced endothelial NO generation [9,11,99,122]. The above-mentioned study further investigated the role of the TRPM2 channel in mediating palmitate-induced inhibition of insulin-induced endothelial NO generation and vasorelaxation [99]. In mouse aortic endothelial cells, exposure to palmitate (500 μM) evoked a cationic current, which was reduced by treatment with TRPM2-specific siRNA as well as by treatment with NAC. Exposure to palmitate also resulted in Ca^2+^ influx that was reduced by treatment with TRPM2-specific siRNA. These results support that palmitate activates the TRPM2 channel via inducing ROS generation. Palmitate, like H_2_O_2_, induced significantly greater currents and intracellular Ca^2+^ increase in aortic endothelial cells from HFD-fed mice than in cells from LFD-fed mice. Palmitate-induced currents and Ca^2+^ responses were reduced in endothelial cells from both LFD-fed and HFD-fed mice that were prior injected via tail vein with adeno-associated viruses expressing TRPM2-shRNA (AAV-TRPM2-shRNA) to deplete the TRPM2 expression. In mouse aortic endothelial cells, exposure to palmitate also inhibited insulin-induced activation of endothelial NO synthase (eNOS) and NO generation. Such palmitate-induced inhibition was alleviated by treatment with TRPM2-specific siRNA and, conversely, intensified by the overexpression of TRPM2. Furthermore, exposure to palmitate induced activation of Ca^2+^/calmodulin-dependent kinase II (CaMKII), which was abolished by treatment with TRPM2-specific siRNA or enhanced by overexpression of TRPM2 [99]. While treatment with insulin alone had no effect, exposure of insulin-treated endothelial cells to palmitate induced activation of PERK kinase, an ER stress signaling molecule, and expression of ATF4, an ER stress-inducible transcription factor, and pseudo-kinase tribble 3 (TRB3), a target of ATF4. These effects were also prevented by treatment with TRPM2-specific siRNA and, conversely, heightened by overexpression of TRPM2. Palmitate-induced activation of PERK, expression of ATF4 and TRB3 and inhibition of insulin-induced activation of eNOS, but not insulin-induced activation of eNOS itself, were prevented by treatment with KN93, a CaMKII inhibitor. Palmitate-induced inhibition of insulin-induced activation of eNOS was also prevented by treatment with 4-phenylbutyric acid, an ER stress inhibitor. Similarly, treatment with insulin led to greater activation of CaMKII and PERK in aortic endothelial cells isolated from HFD-fed mice than in cells from LFD-fed mice and, in addition, induced the expression of ATF4 and TRB3 in cells isolated from HFD-fed mice. These insulin-induced effects in aortic endothelial cells from HFD-fed mice, but not from LDF-fed mice, were mitigated in mice prior injected with AVV-TRPM2-shRNA. Consistently, insulin-induced endothelium-dependent vasorelaxation of aorta from HFD-fed mice, but not from LFD-fed mice, was also significantly improved by treatment with AAV-TRPM2-shRNA. Collectively, these results support that the TRPM2 channel in endothelial cells act as a critical molecular mechanism underlying obesity-associated endothelial insulin resistance [99]; high levels of free fatty acids such as palmitate stimulate ROS generation in endothelial cells to induce TRPM2-mediated Ca^2+^ signaling and activation of the downstream Ca^2+^-dependent CaMKII/PERK/ATF4/TRB4 signaling pathway to inhibit insulin-induced eNOS activation and endothelial NO generation and, therefore, endothelium-dependent vasorelaxation (Figure 5).

### 5.4. Diabetes-Related Oxidative Stress-Induced Alteration of Mitochondrial Dynamics

It is critical for mammalian cells to maintain healthy mitochondrial networks via regulating mitochondrial fission and fusion, or mitochondrial dynamics. In diabetic conditions, the abnormally high level of glucose in blood circulation can induce oxidative stress via excessive mitochondrial ROS generation that can interrupt mitochondrial dynamics and mitochondrial function. Such interruption has been recognized as a molecular mechanism for cellular dysfunction and contributing to the pathogenesis of various later-onset cardiovascular and related diseases [122,123,124,125,126].

**Figure 6:**
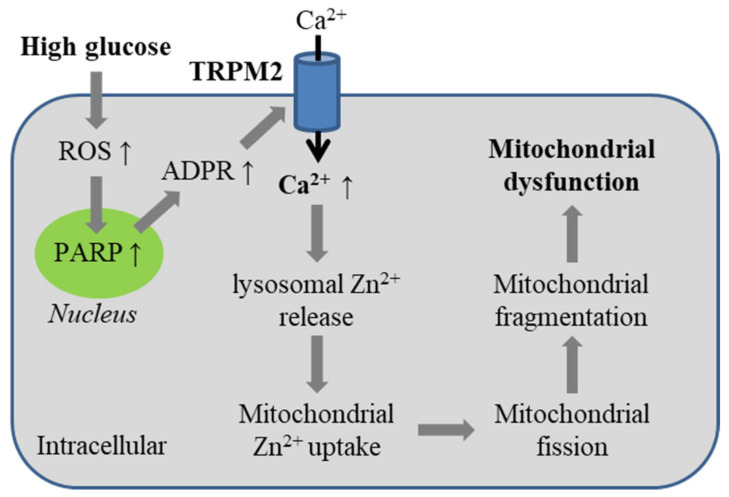
TRPM2-mediated Ca^2+^ signaling in mitochondrial dysfunction induced by high glucose associated with diabetes. Exposure to a high concentration of glucose induces ROS generation and PARP-dependent activation of the TRPM2 channel. TRPM2-mediated Ca^2+^ influx induces lysosomal release of Zn^2+^, and subsequent Zn^2+^ accumulation in the mitochondria recruits Drp-1 to drive mitochondrial fission, leading to mitochondrial fragmentation and dysfunction. Abbreviations: PARP, poly(ADPR) polymerase; Drp-1, dynamin-related protein-1.

A recent study has examined the role of the TRPM2 channel in high glucose-induced alteration in mitochondrial dynamics in endothelial cells [98]. In HUVECs, exposure to H_2_O_2_ (1 mM) for 3 h led mitochondria to change from the healthy elongated and tabular morphology to a morphology characterized by small size and round shape, which was rescued by treatment with TRPM2-specific siRNA or 2-APB. Mitochondria retained the healthy morphology in cells 42 h after cultured in normal medium containing a low concentration of glucose (5.6 mM) but displayed extensive fragmentation in cells growing in normal medium but containing a high concentration of glucose (33 mM). High glucose-induced mitochondrial fragmentation was prevented by treatment with NAC, TRPM2-specific siRNA or 2-APB. Similarly, extensive mitochondrial fragmentation in endothelial cells was documented in mouse lung microvascular endothelial cells and in aortas, which were isolated from WT mice and maintained in high glucose-containing medium. Such effects were largely absent in endothelial cells and aortas from TRPM2-KO mice. These results support a crucial role for ROS-induced TRPM2 channel activation in mediating high glucose-induced alteration of mitochondrial dynamics in endothelial cells (Figure 6).

As further shown using HUVECs [98], high glucose-induced mitochondrial fragmentation was largely prevented by treatment with TPEN, a membrane-permeable and specific Zn^2+^ chelator, but not with chelatordiethylenetriamine, a membrane-impermeable Zn^2+^ chelator, indicating the importance of intracellular Zn^2+^. Likewise, H_2_O_2_-induced mitochondrial fragmentation was prevented by treatment with TPEN or clioquinol, another membrane-permeable Zn^2+^ chelator. Consistently, mitochondrial fragmentation was induced in cells in normal culture medium but with addition of Zn^2+^ and pyrithione (Zn-PTO), a Zn^2+^-specific ionophore, an experimental regime increasing intracellular Zn^2+^ concentration. In HUVECs cultured in normal medium, labile Zn^2+^ was mainly compartmentalized in the lysosomes, not mitochondria or ER, by examining the colocalization of Fluo-Zin3, a Zn^2+^ fluorescent indicator, with a fluorescent marker of lysosome (LysoTracker), mitochondria (MitoTracker) or ER (ER-Tracker). Exposure to H_2_O_2_ (1 mM) for 4 h led to a reduction in the number of LysoTracker-positive lysosomes and Fluo-Zin3-positive vesicles, and an increase in the number of Fluo-Zin3-positive mitochondria. These H_2_O_2_-induced effects were inhibited by treatment with TRPM2-specific siRNA or 2-APB, as well as by treatment with PJ34. Similarly, exposure to high glucose reduced the number of LysoTracker-positive lysosomes and Fluo-Zin3-positive lysosomes, and increased the number of Fluo-Zin3-positive mitochondria, both of which were inhibited by treatment with TRPM2-specific siRNA. In addition, exposure to H_2_O_2_ induced release of lysosomal cysteine proteases into the cytosol, indicating lysosome membrane permeabilization, which was inhibited by treatment with 2-APB. Furthermore, addition of A23187, a Ca^2+^-specific ionophore, in normal culture medium to raise intracellular Ca^2+^ concentration resulted in lysosomal dysfunction and mitochondria fragmentation, both of which were prevented by treatment with TPEN. Induction of these effects by A23187 required noticeably longer incubation, suggesting that intracellular Ca^2+^ induces lysosome dysfunction and lysosomal Zn^2+^ release. It remains possible that intracellular Ca^2+^ also directly affects mitochondrial Ca^2+^ homeostasis, contributing altered mitochondrial dynamics. Exposure to high glucose induced translocation of dynamin-related protein-1 (Drp-1), a small GTPase, from the cytosol into the mitochondria, where Drp-1 works together with mitochondrial fission 1 (Fis1) and mitochondrial fission factor (MFF) to drive mitochondrial fission. As anticipated, high glucose-induced mitochondrial fragmentation was inhibited by treatment with siRNA to deplete the expression of Drp-1, Fis1 and MFF. Translocation of Drp-1 into the mitochondria was also induced by exposure to Zn-PTO. High glucose-induced recruitment of Drp-1 to the mitochondria was prevented by treatment with TRPM2-specific siRNA. Collectively, these results support TRPM2 as an important molecular mechanism for mitochondrial dysfunction in endothelial cells induced by pathologically relevant high glucose. As illustrated in Figure 6, high glucose-induced generation of ROS prompts PARP-dependent TRPM2 channel activation, and TRPM2-mediated Ca^2+^ influx triggers lysosomal Zn^2+^ release and subsequent uptake into mitochondria. Zn^2+^ accumulation in the mitochondria prompts Drp-1 recruitment and drives mitochondrial fission, leading to alteration of mitochondrial dynamics.

## 6. Conclusions, New Questions and Perspectives

The endothelial cell monolayer of blood vessels serves as an important physical barrier to prevent erroneous influx of leucocytes, proteins and proinflammatory mediators to damage the surrounding tissues. It is known that ROS at a modest level acts a physiological signaling molecule regulating endothelial cell function, but the accumulation of ROS to high levels can induce oxidative stress and disrupt endothelial cell function. As discussed above, the TRPM2 channel in endothelial cells has emerged as an important molecular mechanism that mediates ROS-induced Ca^2+^ signaling regulation of endothelial cells. Such TRPM2-mediated Ca^2+^ signaling can be used for a good cause, exemplified by its role in VEGF-induced endothelial cell migration and angiogenesis and, furthermore, post-ischemic neovascularization. However, accumulative evidence suggests that TRPM2-mediated increase in intracellular Ca^2+^ concentration in endothelial cells, induced by various oxidative stress-inducing endogenous and exogenous pathological factors, leads to aberrant activation of Ca^2+^-dependent downstream signaling pathways that impair endothelial barrier function and induce mitochondrial dysfunction and apoptotic cell death. These findings provide new, mechanistic insights into ROS-mediated alterations in endothelial cell function and the pathogenesis of related pathologies.

However, numerous questions are arising and warrant further research. For instance, in high glucose-induced mitochondrial fragmentation, the molecular mechanisms for TRPM2-dependent lysosomal permeabilization and lysosomal Zn^2+^ release, Zn^2+^ uptake into mitochondria and Zn^2+^-dependent recruitment of Drp1 to the mitochondria remain poorly understood. In addition to being a Ca^2+^-permeable channel on cell surface, the TRPM2 channel also functions as a lysosomal Ca^2+^ release channel in pancreatic β-cells, where high glucose-induced lysosomal Zn^2+^ release was shown to critically depend on the TRPM2 channel [92]. There is some evidence to suggest that the TRPM2 channel is present in mitochondria, where the TRPM2 channel is required for mitochondrial Zn^2+^ uptake in neuronal cells [127]. It is interesting to determine whether the TRPM2 channel is expressed in these intracellular organelles in endothelial cells as part of the mechanisms mediating high glucose-induced alteration intracellular Zn^2+^ homeostasis and mitochondrial dynamics. It is also interesting to examine whether TRPM2-dependent Zn^2+^ uptake into the mitochondria is involved in TNF-α-induced apoptosis in endothelial cells. It is unclear whether palmitate, which is related to diabetes as well as obesity, induces TRPM2-mediated mitochondrial fragmentation and mitochondrial dysfunction, as shown for high glucose. In addition, whether such TRPM2-mediated mitochondrial dysfunction promotes mitochondrial ROS generation to form a positive feedback mechanism for further activation of the TRPM2 channel in endothelial cells, as have been proposed in TRPM2-mediated neuronal cell death [127,128]. Intriguingly, TRPM2-mediated Ca^2+^ signaling activates Ca^2+^-dependent downstream signaling pathways, which, albeit seeming not exactly the same, destabilize the inter-endothelial junctions to facilitate VEGF-induced endothelial cell migration and angiogenesis and post-ischemic neovascularization and, also, allow trans-endothelial migration of leucocytes and tissue damage in response to infection. Do these processes occur in vivo in response to different stimuli that all induce ROS generation, and determine the outcomes to be beneficial or detrimental? Finally, more investigations using rodent disease models are required to comprehend the contribution of oxidative stress-induced TRPM2-mediated mitochondrial dysfunction, cell death and disruption of endothelial cell function in the pathogenesis of vascular diseases discussed above. Regardless, the findings raise an interesting perspective on the TRPM2 channel as a new pharmacological intervention of such conditions.
